# Simplified left cardiac sympathetic denervation as an acute strategy for recurrent ventricular tachycardia in multimorbid patients with structural heart disease: A case series

**DOI:** 10.1016/j.hroo.2025.07.009

**Published:** 2025-07-19

**Authors:** Konstantin Krieger, Innu Park, Thomas Kemper, Christoph Lösel, Beate Schädlich, Raphael Spittler, Maren Kirchhöfer, Christina Lohrenz, Stefan Meierling, Boris Alexander Hoffmann

**Affiliations:** 1 Department of Cardiology, Asklepios Klinikum Harburg, Hamburg, Germany; 2 Department of Cardiology, Klinikum Frankfurt (Oder), Frankfurt (Oder), Germany; 3 Department of Electrophysiology, Hospital Braunschweig, Braunschweig, Germany; 4 Department of Cardiology II/Electrophysiology, Center for Cardiology, University Hospital Mainz, Mainz, Germany; 5 Department of Thoracic Surgery, Asklepios Klinikum Harburg, Hamburg, Germany

**Keywords:** Electrical storm, Cardiac sympathetic denervation, Structural heart disease, Ventricular tachycardia, Stellate ganglion

## Abstract

**Background:**

Cardiac sympathetic denervation as a treatment for drug-refractory ventricular arrhythmias (VAs) involves video-assisted thoracoscopic removal of the stellate ganglion (SG) and thoracic ganglia. A simplified approach sparing the SG and targeting left T2–T4 ganglia (left cardiac sympathetic denervation [LCSD]) may offer a less invasive alternative.

**Objective:**

This study aimed to evaluate the efficacy and safety of simplified SG-sparing LCSD as a bailout procedure for multimorbid patients with structural heart disease and recurrent VAs refractory to antiarrhythmic drugs and/or catheter ablation.

**Methods:**

All patients undergoing SG-sparing LCSD at our institution between June 2023 and June 2024 were included in this single-center retrospective study. Baseline demographics, procedural complications, and arrhythmia outcomes were analyzed.

**Results:**

LCSD was performed in 7 patients (mean age 75.9 ± 6.7 years, mean LVEF 30.7 ± 10.9%) with structural heart disease (nonischemic cardiomyopathy, n = 3; ischemic cardiomyopathy, n = 4) mostly 1 day (interquartile range 1–21) after admission with a procedure duration of 20.7 ± 5.3 minutes. Initially, 4 patients (57.1%) had electrical storm. Apart from 1 pleural effusion requiring drainage, no major complications or Horner’s syndrome occurred. During a follow-up of 7 ± 2.6 months, median VA episodes requiring implantable cardioverter-defibrillator therapy decreased from 14 to 2 (*P* = .021) and median implantable cardioverter-defibrillator shocks from 1.5 to 0 (*P* = .034). Three patients remained free of sustained VAs; 1 patient died of coronavirus disease 2019.

**Conclusion:**

In this case series of 7 patients, SG-sparing LCSD demonstrated promising results in terms of safety and efficacy for reducing VAs. Further studies are warranted to confirm long-term outcomes with this approach.

## Graphical abstract

**Figure:**
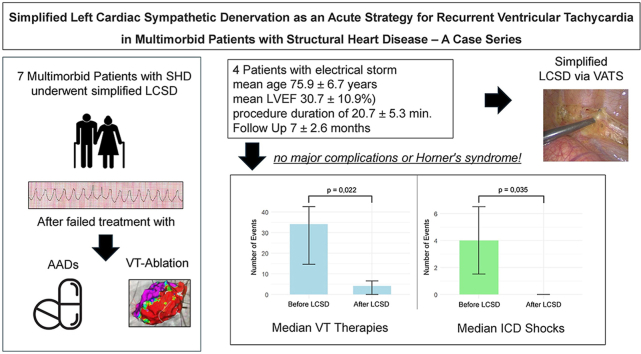

**Key Findings**

▪ Stellate ganglion–sparing left cardiac sympathetic denervation (LCSD) significantly reduced sustained ventricular arrhythmias and implantable cardioverter-defibrillator shocks in multimorbid patients with structural heart disease.
▪ The procedure was well tolerated, with no major periprocedural complications, thus supporting its use in high-risk patients who are not candidates for catheter ablation
▪ Although LCSD significantly reduced the arrhythmic burden, it did not eliminate recurrence in all patients, thus highlighting its role as part of a comprehensive, multimodal treatment strategy.

## Introduction

Refractory ventricular arrhythmias (VAs), including ventricular tachycardia (VT) and ventricular fibrillation (VF), are life-threatening conditions that significantly contribute to morbidity and mortality, particularly in patients with structural heart disease (SHD). Current clinical guidelines emphasize the role of catheter ablation (CA) as a cornerstone in the treatment of these arrhythmias.^1^ However, in progressive disease stages, especially those with extensive myocardial scarring or complex arrhythmogenic substrates, recurrence rates remain high despite CA and antiarrhythmic drug (AAD) therapy.^2^^,^^3^ This underscores the need for alternative therapeutic strategies. The autonomic nervous system plays a pivotal role in arrhythmogenesis, particularly in the context of electrical storm (ES), a condition associated with high mortality and frequently requiring invasive interventions when standard therapies fail.^4^^,^^5^ Neuromodulatory therapy strategies, such as stellate ganglion (SG) block, renal arterial denervation, and cardiac sympathetic denervation (CSD), have emerged as adjunctive or alternative therapies to CA and AADs,^6^^,^^7^ offering a promising approach, particularly in recurrent VAs or ES.^4^, ^5^, ^6^, ^7^ In inherited arrhythmia syndromes, such as long QT syndrome and catecholaminergic polymorphic VT, surgical removal of the left SG and thoracic ganglia T2–T4 (left CSD [LCSD]) has demonstrated efficacy in reducing arrhythmic events.^8^^,^^9^

Both LCSD and bilateral CSD (BCSD) have demonstrated efficacy in reducing VAs and premature ventricular contractions in patients with SHD.^10^, ^11^, ^12^, ^13^, ^14^, ^15^, ^16^ BCSD provides sustained arrhythmia control in long-term studies.^11^^,^^12^^,^^17^ However, in elderly or multimorbid patients, particularly those with ES or incessant VT, CSD may pose procedural challenges and increased risks. To address these challenges, simplified techniques, such as stellate-sparing CSD,^14^^,^^15^ have shown comparable efficacy while reducing complications such as Horner’s syndrome and procedural bleeding. By selectively targeting the left thoracic sympathetic chain, this approach reduces procedural complexity, making it particularly suitable for high-risk patient populations. This study evaluates the efficacy and safety of a simplified SG-sparing removal of left thoracic sympathetic chain T2 to T4 (LCSD) technique in elderly, high-morbidity patients with refractory VA and ES.

## Methods

### Study design

This is a single-center retrospective study that enrolled all consecutive patients with refractory VT who underwent LCSD at Asklepios Klinikum Harburg, Germany, between June 2023 and June 2024. The study protocol was approved by the local ethics committee (Ethics Committee Hamburg, no. 2024-300497-WF) and conducted in accordance with the Declaration of Helsinki on human research. Informed consent was obtained from all participants. Baseline characteristics including drug therapy and echocardiographic parameters were collected. Patients were included in our study if they had refractory VT despite CA or if their medical condition or personal choice precluded CA. In short, CA was performed under conscious sedation using the CARTO 3 electroanatomic mapping system (Biosense Webster) with high-density multielectrode mapping (PENTARAY catheter and NAV ECO mapping catheter, Biosense Webster). Substrate-based endocardial mapping was the primary strategy, especially for hemodynamically unstable VTs. Scar regions were identified by low-voltage areas (<1.5 mV), late potentials, and conduction channels. Ablation was performed using an irrigated catheter at 40–50 W (SmartTouch SF, F-type or D-type, irrigated tip, Biosense Webster), followed by programmed ventricular stimulation (Qubic Stim, Biotronik) for postablation assessment. Refractory VT was defined as sustained VT (sVT) recurrence requiring either clinical therapy or intervention by the implantable cardioverter-defibrillator (ICD), either antitachycardiac pacing (ATP) or shock delivery, despite AAD and/or previous catheter VT ablation attempts. The LCSD was performed after patient assessment by our thoracic surgery unit. The primary efficacy outcome was the recurrence of sVT requiring ICD intervention during follow-up (FU). According to the guideline, sVT was defined as continuous VT of at least 30 seconds requiring intervention to terminate. Incessant VT is defined as continuous sVT that recurs promptly despite repeated interventions to terminate it over several hours.^1^ ES is defined as 3 or more episodes of VT within 24 hours (separated by at least 5 minutes), each requiring intervention for termination.^1^^,^^4^^,^^5^ All patients were followed up clinically, and ICD interrogation was performed at our outpatient clinic every 3–6 months and additionally as clinically indicated. VT recurrence was determined by 3 experienced electrophysiologists (B.A.H., K.K., and I.P.) by review of stored ICD electrograms. ICDs were programmed for secondary prevention in accordance with consensus recommendations.^18^ Detection zones were typically set at 10–20 beats below the clinical VT rate or based on individualized programming if it had been shown to be effective in treating VT. The programming included 2 or 3 therapy zones with delayed detection: VF zone for shock therapy, VT zone (VT/VT2) with ATP and shock, and, when applicable, a slower VT zone (VT1) with ATP. To maintain consistency in arrhythmia detection and therapy, ICD programming was not modified before LCSD or during FU. The secondary efficacy outcome was the reduction of ICD shocks after LCSD. All periprocedural adverse events were evaluated and were defined as any death, hemothorax, pneumothorax, ptosis or Horner’s syndrome, sweating, or other complications occurring within 30 days after the procedure.

### Technique

**Figure 1:**
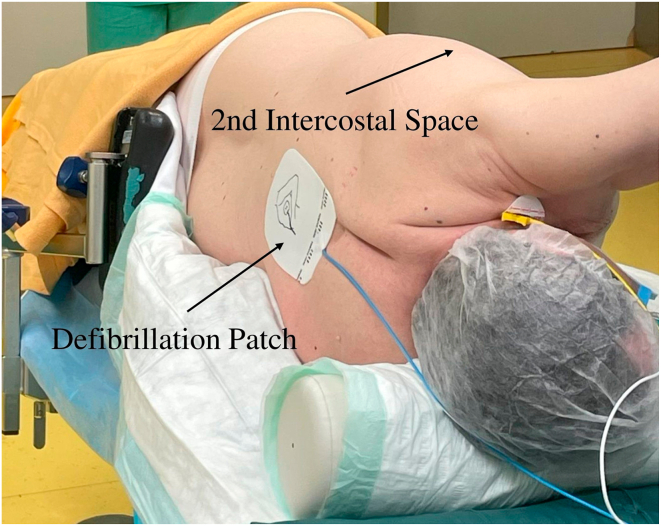
Patient placed in a right lateral position before video-assisted thoracoscopic surgery.

**Figure 2:**
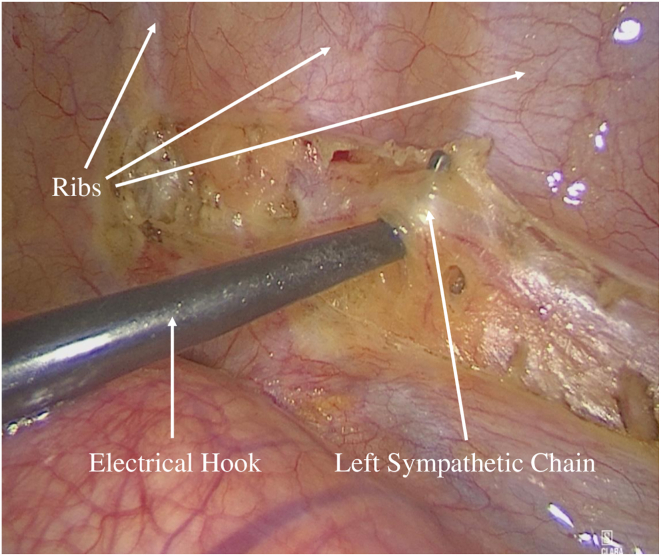
*Left:* Thoracoscopic view illustrating the dissection and elevation of the left sympathetic chain using an electrical hook prior to its removal.

**Figure 3:**
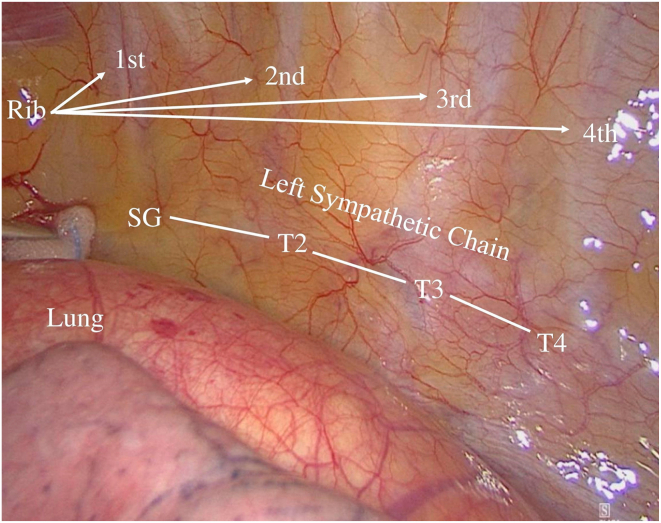
*Right:* Thoracoscopic view of the anatomic landmarks of the left sympathetic before dissection. SG = stellate ganglion; T2, T3, and T4 = thoracic ganglia 2, 3, and 4.

LCSD was performed using a minimally invasive video-assisted thoracoscopic surgery (VATS) approach. Under general anesthesia using a double-lumen endotracheal tube, the patient was positioned in a right lateral position to facilitate optimal access to the axillary region (Figure 1). A small incision was made in the axillary cave at the level of the second intercostal space, through which a 5-mm 30° camera was inserted. In the presence of adhesions or poorly collapsed lung, a second incision allowed the introduction of an electrified hook (5-mm L-Hook Electrode, LaproSurge) configured to 80 W. The sympathetic chain was identified beneath the thin pleura on the posterior chest wall, close to the costovertebral joints. The procedure involved resection and removal of the sympathetic chain from the lower edge of the second rib to the fourth rib, including the thoracic ganglia (T2–T4) and the branching rami communicantes (Figure 2 and 3). Given that ophthalmic nerve fibers typically traverse the upper half of the SG, surgical excision above the second rib was not performed to prevent Horner’s syndrome. The nerve of Kuntz is a common anatomic variant that arises from the second thoracic nerve and may contain sympathetic fibers that bypass the main thoracic chain.^19^ If present, it must be dissected, because failure to do so can result in incomplete denervation. Histologic samples of the thoracic ganglia were taken intraoperatively for postoperative analysis. Pleural drainage (14 ch) was placed at the end of surgery via the axillary approach, based on the surgeon’s judgment. After radiological control, the drainage was removed the evening of the procedure or the following morning.

### Statistics

Descriptive statistics, including the number of observations, mean, and standard deviation, were used to summarize continuous variables. Continuous data were presented as mean ± standard deviation or mean ± standard error of the mean for normally distributed data and median (interquartile range [IQR]) for nonnormally distributed data. Normality of continuous variables was assessed using visual methods (quantile-quantile plots and histograms) and statistical tests (Shapiro-Wilk test). Categorical variables were reported as absolute and percentage values. The Mann-Whitney U test was applied to compare nonnormally distributed variables, whereas paired *t* tests and Wilcoxon signed-rank tests were used to analyze changes before and after LCSD. A *P* value of <.05 was considered statistically significant. All analyses were conducted using R (R Core Team, version 4.4.2) and RStudio (RStudio Team, version 2024.12.0+4670).

## Results

Between June 2023 and June 2024, 7 consecutive patients underwent the simplified LCSD procedure and were included in this study. Baseline characteristics are presented in Table 1, whereas clinical characteristics including VT cycle length, AAD use, ICD programming, and ablation history are presented in Table 2.

**Table 1:** Baseline patient characteristics

Baseline characteristics	
Age (y)	75.9 ± 6.72
Men, n (%)	5 (71.4)
BMI (kg/m^2^)	24.73 ± 3.52
Cardiomyopathy etiology	
Nonischemic, n (%)	3 (42.9)
Ischemic, n (%)	4 (57.1)
Left ventricular ejection fraction (%)	30.7 ± 10.93
Comorbidities	
Arterial hypertension, n (%)	3 (42.9)
Diabetes mellitus, n (%)	3 (42.9)
Coronary artery disease, n (%)	4 (57.1)
Chronic pulmonary disease, n (%)	4 (57.1)
Renal insufficiency, n (%)	4 (57.1)
Atrial fibrillation, n (%)	4 (57.1)
NYHA class	
II, n (%)	3 (42.9)
III, n (%)	3 (42.9)
IV, n (%)	1 (14.2)
Antiarrhythmic drugs	
Amiodarone, n (%)	6 (85.7)
Mexiletine, n (%)	5 (71.4)
Beta-blocker, n (%)	7 (100)

Values are numbers (%), numbers, and mean ± standard deviation.

BMI = body mass index; NYHA = New York Heart Association.

**Table 2:** Clinical presentation including ventricular tachycardia, type of ablation, and antiarrhythmic drugs

Pt no.	Age (y)	Etiology	LVEF (%)	Clinical presentation	VT CL (ms)	ICD programming treatment zones in CL (ms)	VT ablation (number and approach)	Antiarrhythmic drugs	Substrate and ablation	Previous cardiac surgery
1	75	NICM	27	ES	320	VT (1/2): 375/321 VF: 250	No	Amiodarone/mexiletine	-	No
2	80	ICM	25	ES	430	VT: 460 VF: 300	1 × left endocardial	Amiodarone	Apical-basal, lateral aneurysm, RF ablation	2 × ACVB
3	70	NICM	35	ES	588	VT (1/2): 632 /375 VF: 300	1 × left endocardial	Amiodarone/mexiletine	Basal posterolateral LV, RF ablation	No
4	73	ICM	12	sVT	360	VT (1/2): 392/321 VF: 250	1 × left endocardial	Amiodarone/mexiletine	Apical-basal, inferoseptal LV, RF ablation	No
5	70	ICM	30	ES	300	VT: 359 VF: 250	1 × right endocardial	Amiodarone/mexiletine	Inferoapical aneurysm, calcified and fresh thrombus, no left endocardial RF ablation	No
6	89	ICM	43	sVT	380	VT (1/2): 429/319 VF: 250	No	Amiodarone/mexiletine	-	No
7	74	NICM	43	InVT	360	VT (1/2): 370/319 VF: 260	2 × left endocardial	Mexiletine	Basal posterolateral LV, 2 × RF ablation	Mitral valve repair

ACVB = aortocoronary venous bypass; CL = cycle length; ES = electrical storm; ICD = implantable cardioverter-defibrillator; ICM = ischemic cardiomyopathy; InVT = incessant ventricular tachycardia; LV = left ventricle; NICM = nonischemic cardiomyopathy; Pt = patient; RF = radiofrequency; sVT = sustained ventricular tachycardia; VF = ventricular fibrillation; VT (1/2) = ventricular tachycardia treatment zone 1 or 2.

**Figure 4:**
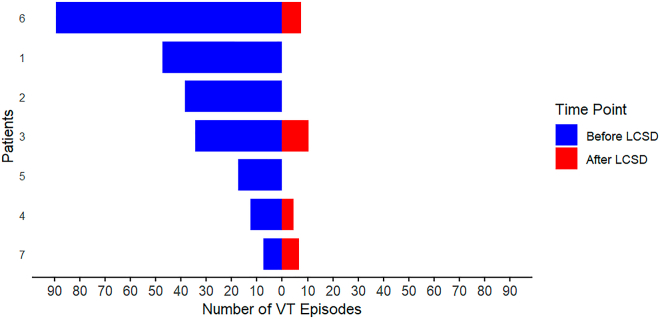
Sustained VT episodes treated by the implantable cardioverter-defibrillator for each patient, comparing the 6-month period before and during follow-up after LCSD. LCSD = left cardiac sympathetic denervation; VT = ventricular tachycardia.

All patients had an implanted ICD, with 3 patients (42.9%) having a dual-chamber ICD and 4 (57.1%) receiving cardiac resynchronization therapy. The cohort exhibited a mean of 1.4 ± 0.53 VT morphologies per patient, with an overall mean VT cycle length of 391.14 ± 96.3 ms (IQR 300–588 ms). ES was documented in 57.1% of cases (n = 4). CA was performed in 5 patients (71.1%), with an average of 0.7 procedures per patient. In 2 patients (28.6%), CA was not performed owing to contraindications such as the presence of left ventricular thrombus formation (n = 1) or patient refusal (n = 1). All patients underwent simplified LCSD under general anesthesia without the need for hemodynamic support, and no VTs requiring intervention occurred during the procedure. Histology was confirmed in all cases. The mean effective procedure time was 20.7 ± 5.35 minutes. There were no major procedural complications. The mean hospital stay was 16.2 ± 11.04 days, and the mean intensive care unit stay was 0.7 ± 0.75 days after LCSD. The median time between the last CA and LCSD was 425 days (IQR 6–2925 days), whereas the median time between the last treated VT and LCSD was 1 day (IQR 1–21 days). During FU, 1 patient developed a sweating disorder in the left hand, and another experienced a pleural effusion requiring drainage 4 weeks postoperatively. No patient developed persistent Horner’s syndrome, although 1 patient exhibited intermittent Horner’s syndrome, which resolved within the first 3 months. Unlike most patients with hyperhidrosis treated with LCSD, our patients did not show compensatory sweating. All patients were successfully discharged, with a mean FU duration of 7 ± 2.58 months (IQR 5–12 months). None of the patients required hospitalization for acute heart failure during the FU period. One patient died of coronavirus disease 2019 pneumonia 5 months after LCSD. The primary efficacy outcome, defined as the occurrence of sVT, was observed in 57.14% of the patients (n = 4), whereas 43.86% (n = 3) remained free of sVT recurrences during FU (Figure 4).

**Figure 5:**
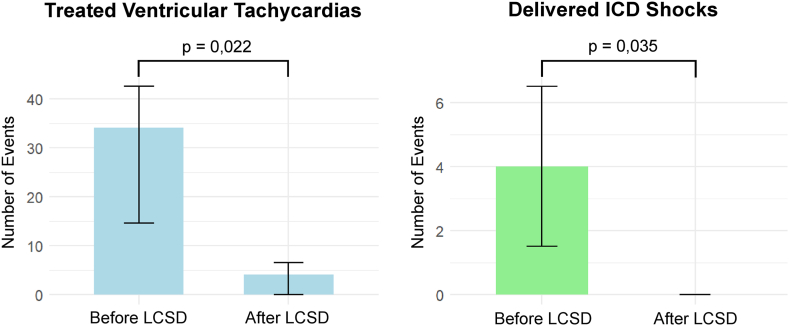
Comparative bar graphs displaying the number of treated ventricular tachycardias (left, *light blue*) and delivered ICD shocks (right, *light green*) during the 180 days before LCSD and the post-LCSD follow-up period. Data are presented as medians with interquartile ranges. ICD = implantable cardioverter-defibrillator; LCSD = left cardiac sympathetic denervation.

After LCSD treatment, the median number of sVT episodes per patient significantly decreased from 34 episodes (IQR 7–89) during the 180 days preceding LCSD to 4 episodes (IQR 0–10) after treatment (*P* = .022). Similarly, median ICD shocks were reduced from 4 shocks (IQR 1–9) before LCSD to 0 shocks (IQR 0–4) after LCSD (*P* = .035), demonstrating a substantial reduction in both sVT episodes and ICD shocks (Figure 5). Only 1 patient received ICD shocks during FU. Two patients (28.57%) experienced early VT recurrence within 1 month, despite continued antiarrhythmic therapy after discharge with amiodarone plus mexiletine or mexiletine plus a beta-blocker. No significant changes were made to beta-blocker or AAD medication during FU. The incidence of the primary efficacy outcome was not significantly influenced by the underlying cardiomyopathy etiology (*P* = 1.0) or presentation with ES (*P* = .42). Furthermore, VT cycle length was not associated with freedom from sVT (*P* = .40).

## Discussion

The results of our study demonstrate the feasibility and safety of a simplified SG-sparing LCSD in reducing the burden of VAs in a multimorbid cohort of patients with SHD. Our key findings are:

1. Simplified LCSD in bailout situations resulted in a significant reduction in VAs and ICD shocks during the FU period (VAs before LCSD vs VAs after LCSD, *P* = .022; ICD shocks before LCSD vs ICD shocks after LCSD, *P* = .035).
2. No major periprocedural complications were observed, highlighting the safety and feasibility of SG-sparing LCSD, particularly in elderly and multimorbid patients who are not eligible for CA.
3. Despite the heterogeneity of the patient cohort, the underlying cardiomyopathy did not seem to influence the outcome of LCSD.

LCSD works by increasing ventricular refractory time and action potential duration, while reducing adrenergic neurotransmitters and has α-adrenergic-receptor blocking properties.^20^ The surgical removal of the left SG was first shown to reduce angina pectoris and arrhythmia in a patient more than a century ago.^21^ Building on this, Schwartz et al^22^ demonstrated in 1976 that left SG block raised the VF threshold and reduced ischemia-related arrhythmias.

Recent studies have demonstrated that thoracoscopic CSD can effectively reduce the burden of VAs in patients with SHD,^11^, ^12^, ^13^, ^14^, ^15^, ^16^ independent of the underlying cardiomyopathy. The heterogeneity of the sympathetic nervous system and its autonomic innervation plays a crucial role in arrhythmogenesis.^23^ The left cardiac sympathetic system, including the left SG, modestly increases heart rate, accelerates atrioventricular conduction, and prolongs the QT interval, primarily innervating the posterior ventricles.^23^ In contrast, the right SG significantly increases heart rate, slows atrioventricular conduction, and predominantly innervates the right ventricle and the anterior regions of both ventricles.^23^ Owing to this heterogeneity, the most performed first-line approach in clinical practice is LCSD, which involves the resection of the lower left SG and T2–T4 ganglia. Although retrospective data show that BCSD may offer improved long-term outcomes in select high-risk patients,^11^^,^^12^^,^^17^ it is generally reserved for refractory cases owing to greater procedural complexity. In the largest study to date(n = 121),^11^ BCSD was associated with longer ICD shock-free and transplant-free survival, but without a significant difference in sVT or ICD shocks compared with LCSD.

Sympathetic cardiac innervation, particularly to the anterior left ventricular wall, is primarily mediated via the left SG; the thoracic ganglia (T2–T4) provide secondary input to the posterior and inferior regions.^23^ The SG’s key role in cardiac innervation is highlighted by the rapid suppression of VAs after its blocking with a local anesthetic.^6^^,^^7^ Nevertheless, Cauti et al^14^^,^^15^ demonstrated that simplified bilateral VATS radiofrequency ablation (sympathectomy) of T2–T4(T5), sparing the SG, is a fast, safe, and effective alternative for treating patients with refractory VT. Our results were comparable concerning freedom from VAs, ICD shocks, and low complication rates with a less invasive SG-sparing LCSD approach.

We adopted a similar approach to minimize procedure duration and reduce the risk of complications, such as postoperative bleeding or persistent Horner’s syndrome, neither of which were observed in our study or the study of Cauti et al.^14^^,^^15^ Although Horner’s syndrome is generally transient and rare (<5%) owing to LCSD,^24^ sparing the SG may further reduce this risk. Surgical removal of the lower part of the SG may increase the risk of bleeding owing to wider resections in this strongly vascularized tissue.^25^ We had no relevant bleeding despite most patients being on anticoagulation and/or antithrombotic therapy. One patient in our case series reported palmar dryness in the left hand, which is a possible side effect of sympathetic denervation. This can occur alongside compensatory hyperhidrosis, which may affect the contralateral side as part of the body’s thermoregulatory adaptation. VATS-assisted removal or ablation of the T2–T4(T5) sympathetic chain is also a well-established procedure in thoracic surgery and is frequently performed as standard therapy for hyperhidrosis.^26^ Waddell-Smith et al^27^ reported that more than 50% of patients experienced palmar dryness after LCSD owing to long QT syndrome and catecholaminergic polymorphic VT. However, most patients still considered the procedure to be successful. This issue should be discussed with patients before the procedure, because it may affect quality of life.

Previous publications have reported favorable outcomes with CSD as a bailout procedure in patients with ES or refractory VT.^13^^,^^16^^,^^17^^,^^28^^,^^29^ In the acute setting of an ES, LCSD has been shown to be sufficient as a treatment option with lower procedural risks than BCSD, particularly in patients with ES who are not candidates for CA, including those on venoarterial extracorporeal membrane oxygenation for hemodynamic support.^28^ However, König et al^13^ highlighted the variability in outcomes with CSD, reporting freedom from VA in only 38.5% of patients and freedom from ICD shocks in 76.9%. Major procedure-related complications occurred in only 2 of 21 patients, but major in-hospital adverse events including transfusion of blood products, infections, and pulmonary embolism were reported in 38.1% of cases.

Despite a comparable freedom from VA and a markedly lower complication rate with an SG-sparing LCSD, the partial efficacy of 43.9% during our FU indicates that an SG-sparing LCSD may not be a sufficient standalone therapy for all patients. Given that most patients had undergone CA and received additional AADs before and during FU, our approach should be incorporated into a broader, multimodal arrhythmia management strategy. The anatomic predominance of posterior and basal arrhythmia substrates in our cohort (Table 2) may have further supported the effectiveness of the LCSD approach sparing the SG. Unfortunately, previous publications by Cauti et al.^14^^,^^15^ describing a similarly simplified approach did not report on the localization of the arrhythmic substrate being ablated or diagnosed, which limits direct comparison. The small size of our case series and the absence of a direct comparison with BCSD may also limit the generalizability of our findings.

Nevertheless, the significant reduction in VA burden and ICD shocks observed in our cohort suggests that this approach may have clinical value as part of a multimodal therapy, particularly for high-risk patients. This strategy was designed to minimize respiratory complications and reduce the need for intensive care unit support, particularly in a hospital with a specialized respiratory unit still dealing with the lingering effects of the severe acute respiratory syndrome coronavirus 2 pandemic.

### Limitations

The study has several limitations that should be acknowledged. First, the small sample size, combined with its retrospective and single-center design, inherently limits the generalizability of the findings. Second, the inclusion of patients with both ischemic and nonischemic cardiomyopathies introduces a degree of heterogeneity that may have influenced the results. Third, the lack of epicardial mapping and ablation represents a methodological limitation, given that this could have had a significant impact on the outcomes. The anatomic predominance of posterior and basal arrhythmia substrates in our cohort may have further supported the effectiveness of the LCSD approach sparing the SG. Furthermore, selection and reporting biases cannot be entirely excluded, given the study’s design. Although the findings provide valuable insights, larger prospective studies are required to validate these results. In particular, a future randomized trial comparing traditional CSD techniques with the SG-sparing approach will be essential to definitively assess their relative efficacy and safety.

## Conclusion

In this small case series of 7 patients, the SG-sparing LCSD method seemed to be a promising acute intervention for multimorbid patients with SHD and recurrent VAs or ES refractory to conventional therapies. This approach was associated with a reduction in both treated VA and ICD shocks during FU, suggesting its potential benefit as part of a multimodal treatment strategy for high-risk patients. The procedure was well tolerated, with no major complications, even in this case series of elderly and multimorbid patients. However, further research is warranted to confirm the long-term outcomes and compare this approach with BCSD and other therapies.
